# Group medical visits in cancer survivorship care: a scoping review

**DOI:** 10.1007/s11764-024-01662-8

**Published:** 2024-11-18

**Authors:** Niharika Dixit, Leslie Avilez, Vlad Honcharov, Kevin Knopf, Teja Bedi, Larissa Nekhlyudov, Urmimala Sarkar

**Affiliations:** 1 University of California San Francisco/Zuckerberg San Francisco General Hospital, San Francisco, CA USA; 2 Division of Internal Medicine, University of California, San Francisco, San Francisco, CA USA; 3 Zuckerberg San Francisco General Hospital, San Francisco, CA USA; 4 Department of Medicine, Alameda Health System - Highland Hospital, Oakland, CA USA; 5 San Mateo Medical Center, San Mateo, CA USA; 6 Department of Medicine, Brigham and Women’s Hospital, Boston, MA USA; 7 Division of General Internal Medicine, Department of Medicine, University of California San Francisco, San Francisco, CA USA; 8 Center for Vulnerable Populations, Department of Medicine, University of California San Francisco, San Francisco, CA USA

**Keywords:** Cancer survivorship, Survivors, Group medical visits, Shared medical visits, Multidisciplinary care

## Abstract

**Background:**

More than 60% of cancer survivors report unmet physical, psychosocial, and informational needs. The care of cancer survivors includes surveillance, health maintenance monitoring, referral for long-term adverse effects of cancer treatment, and coordination of care. Group medical visits (GMV) include medical care, education, and peer support and can be used to facilitate the delivery of multidisciplinary survivorship care. We aimed to characterize the current state of related research describing the role of GMV in cancer survivorship care.

**Methods:**

For this scoping review, we searched for published literature using PubMed, Embase, and other resources. We included intervention studies of multidisciplinary care involving GMVs of adult patients with a history of cancer requiring the presence of medical personnel, physicians, advanced practitioners, or oncology nurses. We included studies that focused on broad cancer survivorship care, rather than those using specific modalities, such as yoga, or limited to specific aspects of cancer survivorship care, such as weight loss. We characterized the studies by cancer type, structure of GMVs, and reported outcomes.

**Results:**

We identified 2311 studies (2122 from PubMed and 189 from Embase). We excluded 1524 duplicates and screened 787 studies for title and abstract review. Finally, 63 studies were retrieved for full-text review, and six were included in this scoping review. Of the included studies, four were non-randomized, and two were randomized. Breast cancer was the most common site (4); other studies included breast and other cancers (1) and hematopoietic transplant cancer survivors (1). There was heterogeneity in the structure and frequency of sessions and the survivorship domains addressed. The outcomes studied included quality of life, healthcare utilization, and costs.

**Conclusions:**

Limited high-quality research exists on the role of GMV in cancer survivorship. Though shown to be effective in chronic disease, the evidence for the effectiveness of this important and widely used approach in multidisciplinary survivorship care remains inconsistent and preliminary; the literature to date provides a starting point for larger-scale studies of GMV in cancer survivorship care.

**Implications for Cancer Survivors:**

While Group medical visits are a promising intervention to provide multidisciplinary care, larger studies are needed to support their benefit in the care of cancer survivors.

## Introduction

Approximately 2 million people are expected to be diagnosed with cancer in the USA in 2023, and the number of cancer survivors is expected to increase to 22.1 million by January 2030 [1]. Survivorship care requires coordination between primary care and cancer care, and it includes surveillance for primary and subsequent cancers, health promotion and disease prevention, as well as monitoring, and referral for late and long-term adverse effects of cancer treatment. Despite multiple published guidelines and recommendations [2, 3], more than 60% of cancer survivors living with cancer or with a history of cancer report unmet supportive care needs in multiple domains, including physical, psychosocial, and informational [4–9]. Notably, unmet supportive care needs are more common in patients from racial and ethnic minority populations [8, 10].

An underlying reason for gaps in survivorship care is the standard care delivery model, which has limited time for patient education during oncological or primary care visits. As care is currently structured, survivors have few opportunities to ask questions during their medical visits [11, 12]. Therefore, the healthcare system must leverage new patient-centered care models to provide efficient, effective, and multidisciplinary cancer survivorship care. These survivorship care models must include comprehensive needs assessment, patient education, and self-management tools to provide comprehensive survivorship care [13, 14]. However, to date, no model has been shown to be optimal in delivering such care [15, 16]. One such intervention for multidisciplinary care is group medical visits (GMVs), also called shared medical appointments (SMAs), a model that has been used in chronic disease management [17, 18]. GMVs allow for longer appointments, provide opportunities to ask questions, and include peer support which may be helpful in the care of cancer survivors [19, 20]. GMVs have demonstrated various benefits for patients with chronic diseases, and while results tend to vary between studies, patients generally benefit from GMVs, which concurrently help alleviate provider burden [21–25]. To what extent GMVs have been used in cancer survivorship care and their associated outcomes is not known. We performed a scoping review of the GMV model for multidisciplinary care in cancer survivorship patients in order to add to the models of cancer survivorship care and to inform future patient-centered interventions of GMV in cancer survivorship care.

## Methods

### Aims and objectives

This scoping review aimed to examine GMVs in multidisciplinary cancer survivorship care for patients treated with curative intent for cancer. We focused on those who have completed treatment and not those who have advanced or metastatic disease due to distinct needs and standards in these two populations. We chose a scoping review rather than a systematic review for two reasons. First, we expected a wide range of study interventions and outcomes, which is more suitable for a scoping review. Second, our objective was to inform the design of a GMV intervention for breast cancer survivors. A scoping review is better suited to describe the heterogeneity of GMV interventions [26]. We chose to narrow our inclusion criteria and focus primarily on broad survivorship interventions to provide a focused and in-depth examination of interventions that have a comprehensive impact on survivorship care. To meet this objective, we analyzed how GMVs are designed and delivered to address the unique needs of cancer survivors.

We defined GMV as defined by Agency for Healthcare and Research Quality where in a group visit patients with a common condition, such as diabetes mellitus, meet as a group in guidance of one or more clinicians and it is part of their regular care [27]. We aimed to assess the characteristics of GMV, including the structure of the GMV, the population of cancer survivors served, the medical personnel involved in the design and delivery of GM, and the inclusion of diverse populations. We used the Quality of Cancer Survivorship Framework [13] to characterize the contextual domains related to the healthcare delivery system, such as clinical structure, communication and decision-making, and patient and caregiver experience. We also identified the domains of survivorship care addressed in the studies, including surveillance for recurrence of cancers and prevention of new cancers, surveillance and management of physical and psychosocial effects, health promotion and disease prevention, as well as chronic conditions. The framework has been used extensively in the systematic evaluations of the literature [15, 28–30].

### Design

This protocol was prepared in accordance with the Preferred Reporting Items for Systematic Review and Meta-Analysis Protocol (PRISMA-P) statement for standardized reporting (see PRISMA-P checklist) and the PRISMA extension for Scoping Reviews (PRISMA-ScR). Though assessment of quality is not required for scoping reviews, we used the Newcastle–Ottawa Scale (NOS) [31] for cohort studies and the Risk of Bias tool for randomized studies [32]. The NOS is a tool used for assessing the quality of non-randomized studies in meta-analyses, focusing on three domains: selection of study groups, comparability of groups, and ascertainment of exposure or outcome. The Risk of Bias tool is a comprehensive assessment tool designed to evaluate the internal validity of randomized controlled trials by examining various domains of potential bias, including selection, performance, detection, attrition, reporting, and other biases.

### Search

We included original research studies (not commentaries or editorials) with primary data that enrolled adults age 18 and older and described or investigated multidisciplinary GMVs in cancer survivorship care survivors delivered by medical personnel, such as physicians, or advanced practice clinicians. We included multidisciplinary interventions that incorporated nursing, social work, and other professionals on teams. We included all formal and informal structures of a multidisciplinary GMV intervention that addressed multidisciplinary survivorship care. We did not exclude based on adherence to the formal structure needed for billing of services. Therefore, included GMV interventions may consist of informal education/management of specific cancer-related topics or may have a formal structure of one-on-one visits, group education components, and documentation in the electronic health record. We prespecified that studies must outline the following components: the population of cancer survivors, the structure of the GMV, and defined outcomes, which may range from feasibility and acceptability to change in knowledge, quality of life (QOL), patient satisfaction, or treatment adherence. We excluded studies that were not cancer-related or were cancer-related but did not focus on survivorship care. We excluded group visit interventions of specific modalities, such as yoga or physical activity, as opposed to the provision of broader survivorship care. We also excluded studies with outcomes that did not focus on comprehensive survivorship care but rather on specific survivorship care-related symptoms, such as insomnia. We conducted the initial search on September 6, 2022, and updated it on April 11, 2023, to include newly published articles.

### Data sources and search strategy

Similar to other scoping reviews of group medical visits [23], we performed searches of large medical literature databases, including PubMed and Embase. We developed search hedges using the following themes: GMV and cancer survivorship. We expanded the cancer themes to include cancer survivors, cancer survivorship, cancer education, cancer treatment, cancer screening, cancer, and neoplasm (Table 1). We expanded the GMV theme to include group visits, group medical consultations, shared medical appointments, and shared medical consultations. The search strategies were outlined using the two databases. We excluded conference abstracts due to lack of information. We also excluded gray literature and limited our search to published studies only. We exported citations from the databases into a bibliography management tool that can be shared between all team members.

**Table 1:** Search strategy with date restrictions

PubMed	(“Cancer Survivors”[Mesh] OR “cancer survivor”[tiab] OR “cancer survivors”[tiab] OR “cancer survivorship”[tiab] OR “Neoplasms/education”[Mesh] OR “Neoplasms/rehabilitation”[Mesh] OR “Neoplasms/surgery”[Mesh] OR “Neoplasms/therapy”[Mesh] OR cancer[tiab]) AND ”Shared Medical Appointments”[Mesh] OR “shared medical appointments”[tiab] OR “group visits”[tiab] OR “group medical visits”[tiab] OR “group visit”[tiab] OR “group medical visit”[tiab] OR “shared medical appointment”[tiab] OR “shared medical consultations”[tiab] OR “shared medical consultation”[tiab])
Embase	(‘cancer survivor’/exp OR “cancer survivor”:ti, ab OR “cancer survivors”:ti, ab OR ‘cancer survivorship’/exp OR “cancer survivorship”:ti,ab OR ‘malignant neoplasm’/exp OR cancer:ti,ab) AND (‘shared medical appointment’/exp OR “shared medical appointments”:ti,ab OR “group visits”:ti,ab OR “group medical visits”:ti,ab OR “group visit”:ti,ab OR “group medical visit”:ti,ab OR “shared medical appointment”:ti,ab OR “shared medical consultations”:ti,ab OR “shared medical consultation”:ti,ab)

### Data screening and selection

**Fig. 1:**
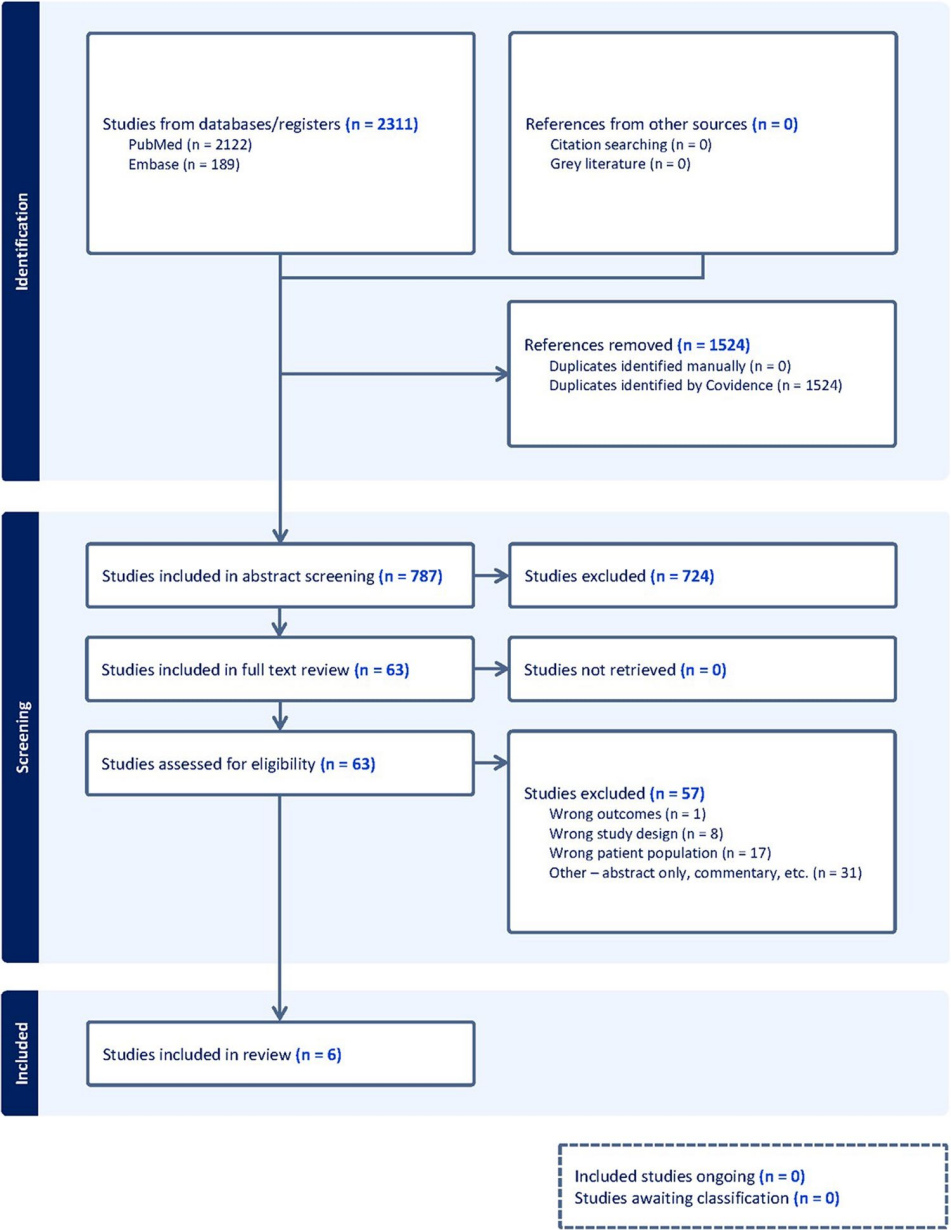
Diagram of study identification and selection process

We used meta-analysis software (Covidence, Melbourne, Australia) to organize and screen the studies. We aggregated the two databases and removed duplicates. First, three team members (ND, VH, LA) independently reviewed and screened article titles and abstracts and selected articles based on inclusion and exclusion criteria while documenting the reason for exclusion. Once we achieved concordance after reviewing 10% of the studies, the two reviewers, VH and LA, reviewed the remaining studies. ND and US reviewed the articles selected for the full-text phase. Next, both reviewers independently reviewed the full texts of the articles, noting the reasons for inclusion or exclusion. Reviewers discussed any discrepancy with ND, and in case of unresolved conflicts, US provided the final determination on the inclusion or exclusion of the articles. We documented reasons for exclusion, and all selection procedures conformed to PRISMA-SCR guidance and are outlined in Fig. 1.

### Data extraction

Two reviewers (VH and LA) independently extracted relevant information on a predeveloped Microsoft Excel (Seattle, USA) form to collect data on study design, population, intervention, control, outcomes, and relevant themes.

### Data analysis and synthesis

**Fig. 2:**
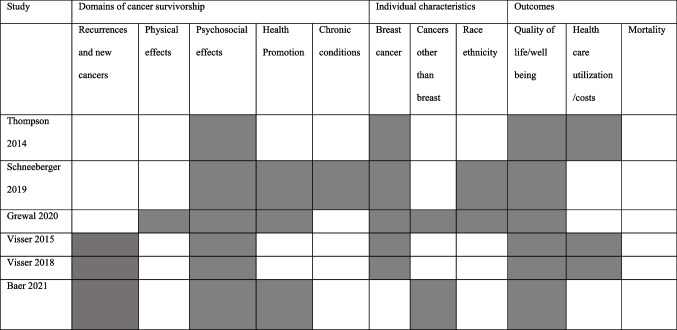
Quality of cancer survivorship care framework components (adapted from Nekhlyudov et al.). The shaded areas represent components included. The unshaded areas represent components not included

We report this scoping review following the PRISMA-SCR format, which includes a checklist of 27 essential items for transparent reporting. In anticipation of heterogeneous, quantitative, qualitative, and observational data, we tabulated it and synthesized it in a narrative format reflecting our study objectives. Figure 2 outlines study details adapted from a framework figure described by Nekhlyudov et al.

## Results

### Search results

We identified 2311 studies (2122 from PubMed and 189 from Embase). After removing 1524 duplicates, we screened the remaining 787 studies for title and abstract review. The abstract screening excluded 724 studies, leaving 63 that were retrieved for full-text review. Following the full-text review, we excluded 57 studies because they did not meet our inclusion criteria for study outcomes (1), study design (8), study population (17), or were in abstract form only (31). Six studies were included in the scoping review.

### Study quality

Of the four non-randomized studies, two were of acceptable quality, with NOS scores of 5 [33] and 6 [34]. The other two studies were of poor quality, scoring two each [35, 36]. The two randomized studies [37, 38] did not have a high risk of bias but had unclear ratings due to the nature of the intervention. Visser [37] relied on self-reported data which can introduce recall bias and confound results. Additionally, the study was underpowered due to a relatively small sample size (*n* = 64). Similarly, Visser [38] also relied on self-reported data.

### Study characteristics

All studies were conducted in Western countries: three in the USA, two in the Netherlands, and one in the UK. The study designs varied, with one mixed methods pilot study with pre- and post-evaluation [33], one retrospective evaluation of a GMV program with analysis limited to participants who had both pre- and post-questionnaires available (21/31) [34], one cross-sectional survey [36] of an ongoing GMV program, one unblinded feasibility randomized controlled trial (RCT) [37], one unblinded multisite RCT [38], and one cross-sectional survey followed by a single-arm intervention [35]. Four studies included only breast cancer survivors [33, 34], although one study included survivors of all cancer types who had completed treatment within 5 years; the majority of participants had a history of breast cancer (58.9%) [36]. Finally, one study included post-hematopoietic transplant (HCT) participants [1]. The mean age of the participants varied from 53.2 to 63.7 years across the studies, while the sample size varied from 7 to 122 participants. The number of participants in group sessions ranged from 4 to 10 across all studies, and the sessions ranged from 90 to 120 min (about 2 h). Only two studies reported race and ethnicity: Baer et al. reported that three participants (4.1%) were Black/African American, 6 (89%) were Caucasian White, and one (1.6%) was Hispanic; in contrast, Grewal et al. reported that 24 (62%) were African American, 14 (36%) were Caucasian, and one (3%) was Hispanic (Table 2).

**Table 2:** Characteristics of included studies

Author and year	Location	Design and duration	Sample size (*N*)	Age (mean)	*n* (%) female	Racial/ethnic background
Visser (2015)	Academic Medical Center, Netherlands	Randomized controlled trial; block randomization, no blinding	*n* = 64; intervention group (*n* = 35); control group (*n* = 29)	Intervention group, 55.03; control group, 57.16	NA	NA
Visser (2018)	One academic and two general hospitals, Netherlands	Multisite randomized controlled trial	*N* = 122; intervention group (*n* = 63) and control group(*n* = 59)	Intervention, 55.8, and control group, 57.9	NA	NA
Thompson (2014)	Sheffield, UK	Mixed methods pilot; 9 months	*n* = 32	63.7	N/A	N/A
Schneeberger (2019)	Wellness Institute, Lyndhurst, OH	Retrospective review; 6-month program	*n* = 21	55.9	N/A	N/A
Grewal (2020)	Academic Medical Center, New Orleans, LA	Cross-sectional survey	*n* = 39	53.2	*n* = 34 (87)	African Americans = 24 (62%); Caucasians = 14 (36%); Hispanics = 1 (3%)
Baer (2021)	Academic Medical Center	Pilot single-arm intervention	*N* = 7	59	Male, 4 (57.1); female, 3 (42.9)	Black = 1; non-Caucasians, 6 (85.7%)

### Theoretical framework

There were significant variations in the structure of the study interventions. Among the included studies, one study was informed by the theoretical framework of Bandura’s social cognitive theory [33] and self-efficacy. Visser et al. [37] were informed by a feasibility approach focusing on demand, acceptability, practicability, cost, integration, implementation, and efficacy, as described by Bowen et al. [39], and finally, Baer et al. were informed by the survivorship framework, described in the Oncology Nursing Society’s Cancer Survivorship: Interprofessional, Patient-Centered Approaches to the Seasons for Survival [40]. Other studies did not report a theoretical basis or framework. The intervention frequencies included weekly [33] and biweekly [20], once only [37], 1 week, 3 months [38], and once every 3 months [35]. The number of sessions varied from a single GMV to seven.

### Contextual factors

The medical personnel conducting the GMV differed in each study. For example, one study included a nurse center manager and an information support worker [33]; however, it implemented an intervention informed by key stakeholders—patients, oncologists, surgeons, clinical nurse specialists, and cancer support center staff. The other two studies included physicians, behavioral health specialists, professional chefs, yoga therapists, dieticians, and social workers. Finally, two studies included only MDs and clinical nurse specialists or social workers [37, 38], and one included physicians, advanced practitioners, social workers, and spiritual health counselors. The GMV structure across studies is outlined in Table 3.

**Table 3:** Structure of GMV intervention

Study	Number of sessions	Duration (minutes)	Personnel	Individual component	Services provided
Visser (2015)	One	90	Oncologist; surgeons; clinical nurse specialist (CNS); social worker	Yes	Breast exam, referrals, prescriptions, and medical record keeping Discussion of both medical and psychosocial themes including breast reconstruction, fear of recurrence, return to work, or lymphedema
Visser (2018)	One in person; 3 optional via app	90	Oncologist; surgeon; CNS; social worker	Yes	Breast exam, referrals Similar to Visser (2015), discussion of both medical and psychosocial themes including breast reconstruction, fear of recurrence, return to work, or lymphedema
Thompson (2014)	Four	120 min	Nurse/ center manager; radiographer/ information support worker; principal investigator (research fellow/counselor)	No	Psychosocial, patient-centered group intervention “Preparing Patients for Discharge” Themes addressed Experience of follow-up. Living with having cancer, threat of recurrence, signs and symptoms, moving on from follow-up
Schneeberger (2019)	Seven	120	Physician; behavioral health coach; dietician; executive chef; yoga therapist	No	Themes addressed Introduction and wrap-up Dietary choices and cooking instruction exercise and yoga, stress relief, breathing exercise, and meditation
Grewal (2020)	(Not specified) ongoing	90–120	Physician; physical therapist; social work; dietician; speech therapist and audiologist (for head and neck patients)	Yes	Themes Discussion of specific side effects including multiple modalities such as aromatherapy, acupressure, and mediation Patients are encouraged to create attainable goals Recommendations for exercise, plant-based nutrition, and mindfulness practices
Baer (2021)	Four		Advance practitioner (AP/physician, spiritual care counselor, and social worker)	Yes	Themes Individual treatment summary, annual preventive care, HCT team information, emotional care, annual preventive care, physical symptoms, treatment factors, supportive services, diet, and nutrition

### Domains of cancer survivorship addressed

None of the studies addressed all domains of survivorship care. We noted variability in the domains of survivorship addressed during the GMV as outlined in Fig. 2. All studies provided services related to the psychosocial domain of cancer survivors, ranging from management of anxiety, chronic stress, mind–body interventions, peer support, and social connections. Visser et al. (2015) conducted a single GMV session called MyGMC, which included individual interaction with clinicians or clinician nurse specialists providing surveillance care focused on recurrence. This was followed by a GMV session facilitated by a social worker and directed by a clinical nurse specialist (CNS) with attention to psychosocial issues. Visser (2018) evaluated the MyGMC intervention further in a randomized multisite study. However, the intervention also included an informational app and three online support groups facilitated by social workers. Two studies also focused on lifestyle-related interventions [34, 36] mapping to the health promotion domain. Baer et al. reported the delivery of survivorship care plans (SCPs) with individual visits followed by group sessions directed toward the emotional, physical, social, and spiritual domains of survivorship for HCT survivors [35]. One study reported preparing participants for discharge from hospital-based care and facilitating the transition from cancer patient to cancer survivor addressing transition of care [33]. The study personnel also addressed the informational needs of the participants on several survivorship care concerns, such as accessing services during the survivorship phase [33], self-care practices and reflecting on the content and goals of the session and integrating them into their daily lives [34], and assessed participants needs and referred to additional services as needed [36]. The details of the sessions in six different studies are included in Table 3.

### Outcomes

The included studies only reported on three distal outcome domains—QOL/well-being, cost, and healthcare utilization, as described below. Patient-reported and objective outcome measures are presented in Table 4.

**Table 4:** Outcome measures and results for included studies

Study	Outcome measures	Reported results
Patient-reported outcomes		
Visser (2015)	• Feasibility • Empowerment—Dutch empowerment questionnaire for breast cancer patients [41] • Cancer worry—cancer worry scale • QOL—EORTC QlQ 30 (European Organization for Research and Treatment of Cancer Quality of Life Questionnaire-Core 30) [42] and EORTC-BR23 (European Organization for Research and Treatment of Cancer Quality of Life Questionnaire-Breast Cancer 23) [43] • Treatment compliance—Medical Adherence Report Scale [44]	No difference in empowerment, cancer worry, QOL, or medication adherence
Visser (2018)	• Primary: psychological distress using symptom checklist-90 [45] • Empowerment using the Dutch Empowerment Questionnaire [41] • Secondary: fear of cancer recurrence, QOL, medication adherence, demand • Feasibility and acceptability • Patient and professional satisfaction • Practicability; self-reported amount time spent in GMC and individual visits • Self-reported usage of app	There was no difference in distress or empowerment between individual or group visits 35% offered participated and 65% declined No difference in worry or QOL Small significant improvement in the medication adherence No difference in satisfaction for patients or professional 78% would consider participation in face-to-face GMC and 32% for app
Thompson (2014)	• Mixed method intervention evaluation • Hospital anxiety and depression scale (HADS) [46]-Anxiety • HADS (depression) [46] • Clinical outcomes for routine evaluation (CORE) [47]—anxiety, depression, well-being, problems, functioning, risk • Measure yourself medical outcomes profile	Participants had a positive experience in terms of their group participation, sharing experience, gaining support and reassurance, and feeling positive about being discharged. No statistically significant differences in psychological assessments were identified, potentially due to small sample size
Schneeberger (2019)	• Perceived stress, depression, patient activation, and QOL • Fat consumption score • Fruits, vegetable, and fiber intake	Changes in psychosocial variables of perceived stress, depression, patient activation, and QOL trended in a positive direction but did not reach statistical significance. Patients reported a significant decrease in average weekly fat consumption (− 31.5%, *p* < 0.01). Most patients found the program educational and enjoyable, and nearly half of them described it as life changing
Grewal (2020)	• Evaluation of SMA, sense of support, improvement in appetite, improvement in pain, loss of weight • Water consumption • Subjective well-being	All patients reported at least some degree of improvement in subjective well-being (SWB). All 39 patients reported that the SMAs helped them in any way; SMAs helped most with sense of support, appetite, weight loss, and pain. Patients who attended > 3 appointments were found to be more likely to report significant/very significant improvement in SWB in comparison to those who attended ≤ 3 SMAs (*p* = 0.03)
Baer (2021)	• Post intervention evaluation by participants	Rated 5 on a scale of 1–5
Objective outcomes		
Visser (2018)	• None	None
Visser (2015)	• Cost of GMC versus individual visit	GMC may be cost-effective if run by social work and clinical nurse specialists
Thompson (2014)	• None	None
Schneeberger (2019)	• Change in: o Weight o BMI o Body fat mass o Lean body mass o Percent body fat	*p* < 0.01 *p* < 0.01 < 0.05 < 0.01
Grewal (2020)	• None	None
Baer (2021)	• Feasibility	Did not meet target accrual of 10 patients over 1 year None of the patients completed all 4 sessions

### Health-related quality of life/well-being

There was significant variability in outcomes, and two studies included an evaluation of the intervention itself [33, 36]. All studies reported health-related QOL/well-being-related outcomes, including psychological measures such as depression, anxiety, and perceived stress [33, 34], as well as assessments such as the cancer worry scale, and QOL measures, symptom checklists [37], and subjective well-being (SWB) [36]. Other patient-reported outcomes included medical outcomes and patient activation [34]. Only one study reported improved SWB, with participants who attended > 3 appointments more likely to report significant or very significant differences in SWB [36]. One study reported changes in dietary intake with unchanged pre-post weekly fruit, vegetable, and fiber consumption [34] but a significant difference in fat consumption score (< 0.01) [34]. One study also included qualitative analysis of free text comments and conducted qualitative interviews [33]. Participants in the included studies found the GMV helpful and reported a greater sense of support and improvement in appetite, pain, and weight loss [36]. One study reported the role of shared experience in reducing feelings of isolation and gaining support from facilitators and fellow survivors [33]. Although group dynamics were not formally evaluated, one of the studies included participants’ reports of a sense of support from providers and fellow cancer survivors [36]. None of the studies included an evaluation of social isolation.

Objective measures were assessed and reported by only one of the studies [34]. The outcomes included body composition, weight, body mass index (BMI) changes, body fat mass, lean body mass, and percent body fat. There were statistically significant weight changes (*p* < 0.01), BMI (< 0.01), body fat mass (< 0.05), and lean body mass (< 0.01) [34] with no difference in percent fat. No studies reported on mortality outcomes.

### Healthcare utilization/costs

From a healthcare utilization standpoint, one randomized study reported that the cost was greater for the GMV than for individual visits if medical specialists participated in the GMV. The cost was lower when a clinical nurse specialist (CNS) and a social worker participated [37]. One non-randomized study reported that participants’ medical insurance was sufficient to cover program costs and attendance at the GMV [36]. A study conducted in a country with a national health program with limited resources reported that the intervention reduced the number of visits to hospital-based care from 12 visits over 5 years to three visits over 5 years [33]. Finally, among the HCT participants, the study was able to enroll only seven participants from a target sample size of ten participants, and none of the participants completed all the sessions due to hospitalization/death, a decline in health, transportation, or other undisclosed reasons.

## Discussion

This scoping literature review summarizes the current published literature on GMV in survivorship care. Despite their demonstrated benefit in multiple chronic conditions, including diabetes [24], prenatal care [17], and other chronic health conditions, such as hypertension [18], pelvic pain [48], and cardiovascular disease [18], the role of GMV in cancer survivorship remains undefined. This review found six studies that evaluated multidisciplinary GMV, with only two being randomized controlled studies. We observed significant variability in the structure of the GMV utilized across studies and domains of cancer survivorship. Interestingly, all studies addressed the psychosocial domain of survivorship care while a few addressed surveillance for cancer recurrence, physical effects, and health promotion. None of the studies addressed chronic conditions, a key aspect of survivorship care. These findings are consistent with the prior systematic reviews of the literature [15, 28–30]. Within the contextual factors, there was heterogeneity in the structure of the interventions, and only Visser (2018) included interpersonal context in the form of peer support or group dynamics. These studies included QOL/well-being, cost, and healthcare utilization outcomes but did not assess mortality. While both the RCTs showed no difference in primary outcomes between the GMV arm for distress and empowerment, Visser (2018) reported higher peer support and satisfaction in the GMV arm [38].

Similar to the prior studies of the GMV in non-cancer conditions, these studies provide preliminary evidence of the role of psychosocial and peer support in managing pain and improving QOL [20, 48, 49]. As outlined in prior research, they also provide preliminary support for GMV in self-management of surveillance, symptoms, and healthy lifestyle changes [50]. In contrast with GMV in non-cancer conditions [29, 31], studies included in this review provide only preliminary evidence of improved patient experience and do not provide definitive evidence of improved outcomes GMV in survivorship care. Finally, group intervention in cancer care has shown promise in single modality or symptom-based approaches that are beyond the scope of this review. These interventions include integrative medicine [51], exercise interventions [52], and symptom management [53], supporting the role of GMV in delivering multidisciplinary survivorship care.

Taking into consideration the evidence from non-cancer survivorship research, our scoping review highlights the need for larger randomized studies comparing well-designed, stakeholder-informed GMV interventions to usual care in the context of cancer survivorship. Investigators should design GMV interventions to address different domains of cancer survivorship with longer follow-up periods and to evaluate outcomes such as quality of survivorship, health-related quality of life, cost, healthcare utilization, and cancer-specific and overall mortality. In addition, future research should evaluate group dynamics and peer support to assess how such interventions can maximize benefits to participants.

There are several limitations to this scoping review. The search identified six studies of modest size, with varying study designs and cancer types. The lack of race/ethnicity data limits our ability to assess the generalizability of the studies to diverse patient populations. Not all studies were high-quality, and thus, their conclusions are subject to bias. Some studies evaluated only an ongoing program [34, 36] and had missing data, possibly introducing bias. One study reported selection bias in which participants from a prior study were invited to participate, and the majority declined participation (140/172) [33]. As expected from the smaller pilot studies, all included interventions were brief, with short follow-up periods, and lacked longitudinal data. Finally, all the studies relied on patient self-reports for most outcome measures. Furthermore, all the studies were small, had methodological limitations, and exhibited limited external validity due to study design, limited follow-up, and lack of theoretical or framework-informed interventions. Primary care is integral in the care of cancer survivors and was not included in these studies. Finally, all studies were conducted in high-income countries, and only two reported race and ethnicity further limiting generalizability. It is possible that some GMV interventions in practice remain unpublished and could not be included in the review. Despite these limitations, this review represents a step forward in designing GMV for cancer survivors.

## Conclusions

This scoping literature review revealed that the current evidence remains limited. With an increasing number of cancer survivors and current unsustainable healthcare models, patient-centered care models such as GMV/SMA can serve as a supplementary or alternative approach to managing survivorship care, but high-quality empiric evidence is needed.

## Data Availability

No datasets were generated or analysed during the current study.
